# The impact of background music on film audience’s attentional processes: Electroencephalography alpha-rhythm and event-related potential analyses

**DOI:** 10.3389/fpsyg.2022.933497

**Published:** 2022-11-17

**Authors:** Young-Sung Kwon, Jonghyun Lee, Slgi (Sage) Lee

**Affiliations:** ^1^Department of Media and Communication, Dong-A University, Busan, South Korea; ^2^Department of English Language and Literature, College of Humanities, Seoul National University, Seoul, South Korea; ^3^Department of Media and Communication, Pusan National University, Busan, South Korea

**Keywords:** background music, tempo, film music, attentional processes, EEG, ERP, alpha-rhythm suppression

## Abstract

Background music is an indispensable part of films and plays an important role in enhancing audiences’ attention to scenes. However, few studies have examined the cognitive effect of background music at the neurophysiological level. Using electroencephalography (EEG), the present study examines the effect of background music tempo on the viewer’s attentional processes. Participants’ (*N* = 24) EEG responses were recorded while the participants watched segments of action films in three conditions with variations on the presence and tempo of background music (i.e., no background music vs. slow-tempo music vs. fast-tempo music). These responses were analyzed using the alpha-rhythm suppression and event-related potential (ERP) P300, a brainwave indicator of attentional processes. The results suggest that participants’ attention levels increased when background music was present (compared to when background music was absent), but there was no difference in participants’ attention levels based on tempo. The theoretical and practical implications of these findings are discussed.

## Introduction

Sound is an important element of entertainment media. For instance, well-designed sound effects or background music in a film can positively influence audience engagement in that film. In films, sound consists of background music, sound effects, and characters’ dialog. In combination with visual components (i.e., video footage), sound contributes to forming the overall mood of a film and inducing emotional responses from the audience (Holman, 2012). In particular, background music can influence viewers’ cognitive processes involved in perceiving and learning from visual components of the content (Kämpfe et al., 2011; Du et al., 2020). For instance, research suggests that people better recall visual information they acquire while being exposed to background music (Proverbio et al., 2015; Nguyen and Grahn, 2017). Background music can also help individuals immersed in the plot of multimedia content (Zhang and Fu, 2015) and induce motor resonance, a neurophysiological indication that viewers are immersed in the action performed by characters in films (Kwon et al., 2021).

As background music is considered an important factor in audience engagement, sound designing—creating and inserting various sound elements in media content—has become an indispensable part of film production. Research in this area has examined how producers use various sound elements to draw and maintain viewers’ attention (e.g., Mitchell, 2005; Holman, 2012); however, relatively little is known about whether such use of sound elicits high levels of attention among the audience. Moreover, in the few studies that have examined the effects of background music on viewers, self-report data (*cf.* Kämpfe et al., 2011; Zhang and Fu, 2015; Lee et al., 2020) have predominantly been used. However, self-report data are limited in terms of objectively capturing subtle and subconscious changes in individuals’ attentional processes that occur in response to exposure to a series of visual and auditory stimuli (Kwon et al., 2021). Instead, neurophysiological methodologies such as electroencephalogram (EEG) are required to directly measure and quantify participants’ cognitive processes. Hence, the present study uses neurophysiological data from EEG to examine the impact of background music on viewers’ attentional processes.

Several studies to date have used EEG to explore the effect of background music on attentional processes (Iwaki et al., 1997; Du et al., 2020; Ausin et al., 2021; Uhm et al., 2021). Most of these studies have explored how background music affects listeners’ attention during various activities or situations, such as while studying, resting, or watching an advertisement. However, studies examining how background music affects the audience of *video media*, particularly film, are scant. It should also be noted that different types of background music—depending on when or where they are used—serve different functions and purposes. For instance, background music played during studying might be considered as noise or “background” music, as the music is not so relevant to the foreground activity (i.e., studying). In contrast, background music in film is pre-selected and is closely tied to the video. Background music inserted in advertisements also has a specific purpose: to induce the audience to remember the product and eventually purchase it, which is quite different from the purpose served by music used in films. Therefore, it is difficult to generalize the prior findings to the context of film background music. Furthermore, measuring attentional processes of film audiences using EEG can offer useful information and a practical guide for optimizing sound-designing in film content production.

The present study particularly focuses on the *tempo* of background music and examines whether various degrees of tempo (slow vs. fast) elicit different levels of attention among viewers. As a basic element of music—along with tonality, range, intensity, and instrument—tempo helps form the overall mood of a piece of music. Tempo is also known to influence the listener’s behavior and affect; music with a fast tempo evokes positive-valence emotions such as happiness, whereas slow music triggers negative emotions such as sadness (Gagnon and Peretz, 2003). Music tempo can also affect the tempo of the listeners’ activities that are being performed while the listeners are exposed to music (Kämpfe et al., 2011).

Relatedly, the tempo of background music plays an important role in setting the ambience of a scene. In film production, music with different tempos is strategically used to produce different moods. For example, for scenes depicting high-tension activities, such as fighting or running, fast music is inserted to convey the tension of the activities, whereas slow music is often used when producers need to create a soft and relaxing ambience. This is based on the conventional notion that the audience will be more absorbed in the scene when the rhythms of activities portrayed and the background music are harmoniously matched. However, there is no empirical evidence, to our knowledge, to suggest that such a match in tempo would lead to a high level of attention among the audience.

Moreover, prior research offers mixed findings about how tempo can affect listeners’ cognitive activities. As noted, studies have found that tempo can determine listeners’ emotions and the pace of their actions. For instance, fast music can induce upbeat emotions (Liu et al., 2018a,b) and fast-paced behaviors (Kämpfe et al., 2011). This indicates that a fast tempo can result in some degree of stimulation in listeners’ cognitive activities. Other studies, however, suggest that fast-beat background music is more detrimental than slow music to the cognitive processes involved in perceiving and evaluating visual information (Kallinen, 2002). Such conflicting findings on tempo make it difficult to predict how background music with different tempos may affect viewers’ attentional processes toward films.

Based on this set of considerations discussed, the present study explores whether (a) the presence of background music and (b) its tempo can enhance viewers’ attention to film scenes. Specifically, we ask the following questions: (a) Does the *presence* of background music lead to an increase in attentional processes toward film scenes?; (b) How does the *tempo* (fast vs. slow) of background music influence the viewers’ attentional processes toward film scenes?

To measure attentional processes, the present study used EEG. Through the use of small metal discs (electrodes) attached to an individual’s scalp, EEG measures the electrical impulses generated from the transmission between nerve cells when the individual is engaged in a cognitive activity. In this study, EEG was employed rather than other neurophysiological methodologies, such as magnetoencephalography (MEG), functional magnetic resonance imaging (fMRI), positron emission tomography (PET), and computer tomography (CT), because EEG is superior in measuring *real-time* brain activities, with relatively less obtrusion and interference in subjects’ physical activity (Mulert and Lemieux, 2009). EEG is known as having excellent temporal resolution—measuring an instantaneous brain cognitive processing response in 0.001 s—and is considered a suitable method for analyzing information processing that takes place rapidly in real time, such as image recognition (Sanei and Chambers, 2013; Cohen, 2014). EEG is, therefore, appropriate for an experiment that requires the recording and detecting of immediate changes in participants’ cognitive processes when participants watch a stream of visual (e.g., film footage) and auditory stimuli (e.g., background music; Kwon and Lee, 2020).

Specifically, we used alpha-rhythm suppression and event-related potentials (ERP) as indices to measure the attentional process. Alpha-rhythm inhibition, measured at 7–13 Hz, is known to be an indicator related to the attentional process. This can be used as an indicator of how much the audience pays attention to an image (Kwon and Lee, 2020). This study also used event-related potentials (ERP) analysis, which is a commonly used EEG in speech-processing research in the field of cognitive science. ERP uses a type of EEG that is obtained by presenting an auditory stimulus to a subject and measuring their brain activity at that moment. P300, one of the ERP elements, was used as an index to measure participants’ attention level (Kwon and Ha, 2019).

## Materials and methods

### Participants and procedure

A total of 24 right-handed participants (10 women, 14 men) were recruited from a university in Seoul, South Korea. The average age of the subjects was 23.04 years (*SD* = 2.94). The number of participants was determined based on the power analyses (Sig. level = 0.05, power = 0.95, effect size = 0.3), using previous studies that used EEG under similar conditions. The experiment has been approved by the Internal Review Board of one of the authors’ affiliated universities.

The experiment was conducted in a sound-proof laboratory that blocks external electromagnetic waves and noise. Upon participants’ arrival, they were ushered to a sound-proof laboratory and seated in front of a desk with a 21-inch monitor on which stimuli footage would be shown. Electrode gel was then applied to the participants to help the sensors stick to the participants’ scalps more easily. Ten-minute long stimulus footage was presented to each participant using PsychoPy3. Participants wore EEG gear throughout the experiment while they watched the stimulus footage. At the beginning of the experimental session, all participants were instructed to pay attention to each scene and minimize their eye blinking and body movement while watching the stimuli.

### Stimulus

The stimuli footage used in the experiment consisted of 45 action scenes (edited from 26 Korean and international action films) in which one subject strikes another. These action (striking) scenes were selected for the following reasons: (1) the action scenes involve large movements in action and were therefore expected to induce a high level of attention, especially at the moment of striking; (2) the clear display of the target behavior (striking) in these scenes creates a concrete point to which participants can fixate their attention. These make action scenes suitable for measuring alpha-rhythm inhibition and event-related potentials (ERP), indicators related to the attentional process. The action scenes also included various motions (i.e., striking with fists, kicking, etc.), with different numbers of characters involved in the actions (two, three, or more people), various framing shots (i.e., close-up, medium shot, and long shot) and shooting techniques (i.e., time-lapse, high-speed shooting, etc.) to avoid bias that may occur from using a particular motion or technique only.

The sequence of these scenes was designed using a counter-balanced paradigm (Goodwin, 2009; Burns and Dobson, 2012; Stangor, 2014). Within a counter-balanced paradigm, each participant is exposed to all treatment conditions where each condition is presented in different orders. A counter-balanced paradigm thus prevents the order effect in which the order of image stimulation affects the dependent variable. The paradigm can also reduce the bias that may occur due to participants’ individual differences. Counter-balanced paradigm is therefore commonly used in neurophysiological studies that use two or more experimental conditions (e.g., Goodwin, 2009).

**Figure 1:**
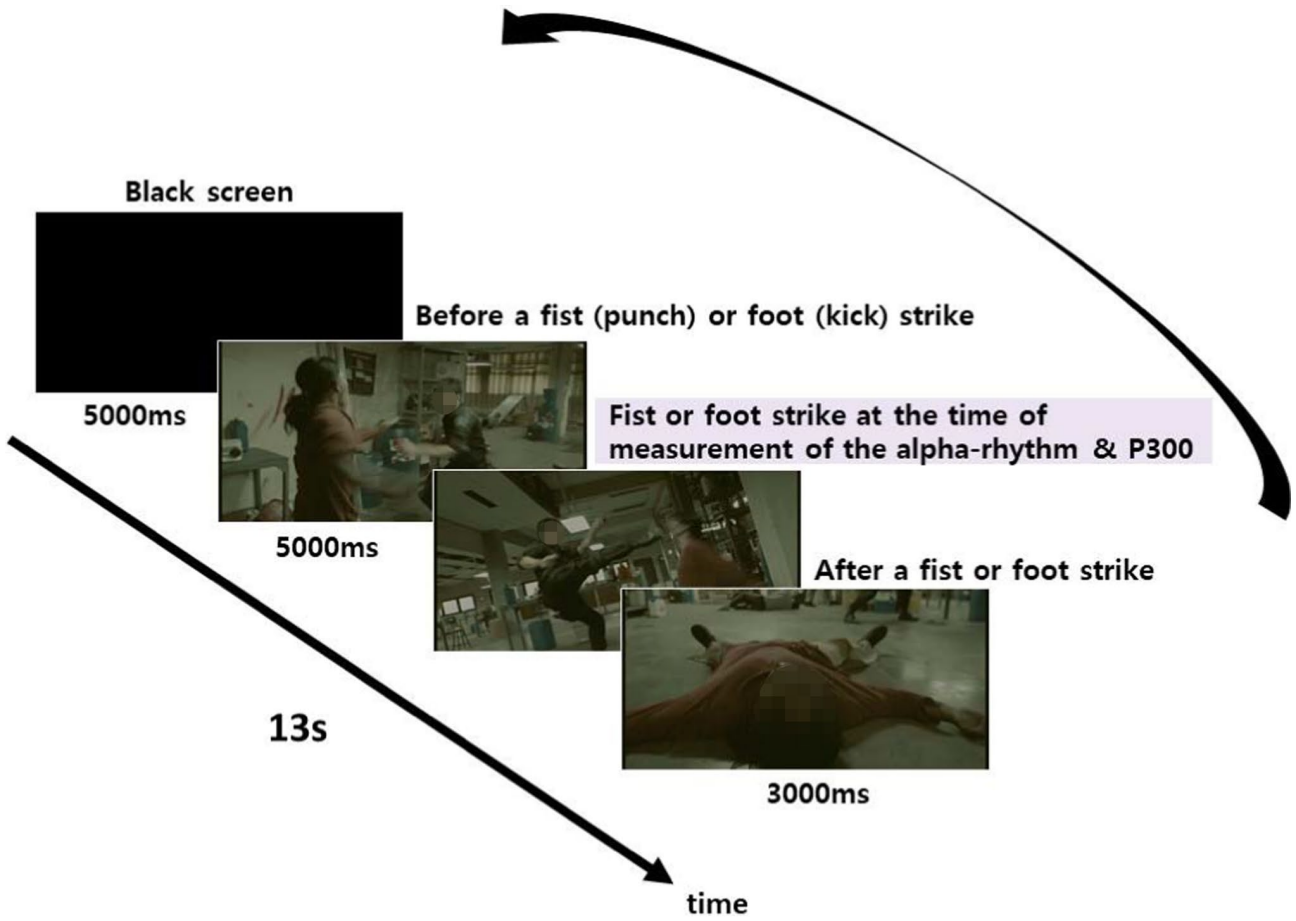
Video composition for individual stimuli.

The stimuli footage used in the experiment consisted of 45 action scenes, edited from 26 films. Three different versions (no background music, slow-tempo music, fast-tempo music) were made from each scene; therefore, a total of 135 scenes (45 scenes × 3 versions) were produced. Three different sets of sequences were then created by randomly ordering the 135 scenes. Each sequence set included 45 different scenes and was assigned to either Group A, B, or C. Participants randomly assigned to one of the three groups were thus shown one of the sequences. Each scene in the sequence was 8 s-long, and 5 s-long black screens were shown between each scene to measure each participant’s baseline (see Figure 1). The sequence of the scenes shown is presented in Table 1.

**Table 1:** The sequence of the counter-balanced stimulus presentation.

Order	**1**	**2**	**3**	**4**	**5**	**6**	**7**	**8**	**9**	**10**	**11**	**12**	**13**	**14**	**15**	**16**
Group A	N-M (1)	S-M (16)	F-M (31)	F-M (32)	N-M (2)	S-M (17)	F-M (33)	F-M (34)	F-M (35)	S-M (18)	S-M (19)	N-M (3)	F-M (36)	F-M (37)	S-M (20)	N-M (4)
Group B	F-M (1)	N-M (16)	S-M (31)	S-M (32)	F-M (2)	N-M (17)	S-M (33)	F-M (3)	S-M (34)	S-M (35)	N-M (18)	F-M (4)	F-M (5)	S-M (36)	N-M (19)	F-M (6)
Group C	S-M (1)	F-M (16)	N-M (31)	S-M (2)	F-M (17)	F-M (18)	N-M (32)	S-M (3)	S-M (4)	N-M (33)	F-M (19)	S-M (5)	S-M (6)	N-M (34)	F-M (20)	S-M (7)
	**17**	**18**	**19**	**20**	**21**	**22**	**23**	**24**	**25**	**26**	**27**	**28**	**29**	**30**	**31**	**32**
	S-M (21)	S-M (22)	N-M (5)	F-M (38)	S-M (23)	F-M (39)	N-M (6)	F-M (40)	N-M (7)	S-M (24)	F-M (41)	F-M (42)	S-M (25)	N-M (8)	S-M (26)	F-M (43)
	N-M (20)	S-M (37)	F-M (7)	F-M (8)	N-M (21)	S-M (38)	F-M (9)	S-M (39)	F-M (10)	N-M (22)	S-M (40)	F-M (11)	F-M (12)	N-M (23)	S-M (41)	F-M (13)
	F-M (21)	N-M (35)	F-M (22)	S-M (8)	F-M (23)	N-M (36)	F-M (24)	S-M (9)	F-M (25)	F-M (26)	N-M (37)	S-M (10)	F-M (27)	F-M (28)	N-M (38)	S-M (11)
	**33**	**34**	**35**	**36**	**37**	**38**	**39**	**40**	**41**	**42**	**43**	**44**	**45**			
	N-M (9)	S-M (27)	S-M (28)	N-M (10)	S-M (29)	N-M (11)	F-M (45)	N-M (12)	N-M (13)	F-M (44)	N-M (14)	S-M (30)	N-M (15)			
	S-M (43)	N-M (30)	N-M (25)	F-M (15)	F-M (14)	N-M (26)	N-M (27)	S-M (44)	N-M (28)	N-M (24)	S-M (45)	N-M (29)	S-M (42)			
	N-M (39)	S-M (12)	N-M (40)	S-M (13)	N-M (41)	F-M (29)	N-M (42)	F-M (30)	N-M (43)	S-M (14)	N-M (44)	S-M (15)	N-M (45)			

The number in parentheses = selected stimulus number. N-M = No Music, F-M = Fast tempo music, S-M = Slow tempo music.

To accentuate the moment where striking action occurs in the scenes, the striking sound effect was uniformly inserted in all scenes. Any other sound or sound effect was removed in all conditions. The background music inserted in the slow- and fast-tempo conditions was composed using the same drum solo sound, but it differed in its tempo (slow vs. fast tempo). The purpose of using a drum solo was to eliminate the influence of other musical elements than tempo, such as tonality and range. The beats per minute (BPM) for the two types of background music used in the slow- and fast-tempo conditions were 144 BPM (fast tempo) and 48 BPM (slow tempo), respectively classified as *allegro* (fast, BPM = 120 ~ 168) and *largo* (slow, BPM = 40 ~ 60) in prior studies (Palit and Aysia, 2015; Fernández-Sotos et al., 2016; Liu et al., 2018a,b).

### Electroencephalogram analysis

#### Electroencephalography recordings

Electroencephalogram was recorded from 32 Ag/AgCl electrodes mounted in an ActiCap (Brain Products GmbH, Germany), arranged following the 10/20 system, using BrainVision Recorder (BrainProducts GmbH). The activities of the alpha rhythms have traditionally been linked to the attentional process of the brain (Klimesch, 2012), and overall suppression in alpha power has been linked to an increase in attention in general (Klimesch et al., 1997; Sauseng and Klimesch, 2008). Alpha-rhythm suppression originally appears in the occipital lobe, frequency distribution of 8–13 *Hz*, and it is actively observed at the O1 and O2 electrodes (Berger, 1929; Fu et al., 2001; Perry et al., 2011). Among the electrode sites, O1 and O2 located in the occipital lobe were used in the present analysis as the alpha rhythm measurement range. The EEG data of each participant was segmented separately among the three conditions followed by a baseline correction and average calculation for each condition. By subtracting the baseline from each condition, three difference waves and the grand average values were obtained for each participant’s data.

Along with the alpha-rhythm suppression, an ERP element named P300 was used to evaluate participants’ attention function. P300 appears in the process of changing the cognitive model, which was established for the subject to successfully respond to a specific task (Polich, 2007). What is important is that the brain becomes involved in attention-related cognitive function in this process (Polich, 2003; Sur and Sinha, 2009). Thus, whether the subject paid attention to the corresponding experimental stimulus can be detected by measuring P300, and how much the subject concentrated on the stimulus can be explored through the amplitude of ERP. P300 occurs in a positive electrode of 200 ~ 400 ms after the stimulus is presented (Hillyard and Kutas, 1983; Picton, 1992), and it is actively observed at the Fz and Cz electrodes located in the frontal and central lobe (Polich, 2003, 2007; Polich and Criado, 2006). The present analysis therefore focused on Fz and Cz in the time windows between 200 and 400 ms to measure P300.

Additional electrodes (vertical electrooculogram and horizontal electrooculogram) were attached next to and below the eyes to detect noises caused by eye activities, such as blinking. All electrode impedances were maintained below 10 kΩ. Extracted signals were amplified through actiCHamp (BrainProducts GmbH) and digitized at a sampling rate of 500 Hz.

#### Pre-processing and statistical analysis

BrainVision Analyzer 2.0 (BrainProducts GmbH) was used for pre-processing. EEG data was off-line re-referenced to average mastoids and filtered with a bandpass filter (a low cutoff of 0.5 Hz and a high cutoff of 40 Hz). Eye blinks and horizontal saccades were corrected semi-automatically using the Independent Component Analysis (ICA) procedure with infomax algorithm implemented in the BrainVision Analyzer. The data were segmented in the range of 0 to 1,000 ms from the point at which a punch or kick touches the opponent. The baseline was corrected in the range of –200 to 0 ms. To extract ERPs, segments were averaged independently in each participant for each condition and then grand-averaged.

For the alpha-rhythm analysis, time-frequency analysis *via* Morlet wavelet transformation in the frequency range of 5–30 Hz was conducted. In doing so, Brain Vision Analyzer 2.0 (Brain-Products GmbH) was used to extract the alpha rhythm from the EEG signal. Wavelet transformation is one of the frequency analysis methods that can concurrently visualize time, power, and frequency, and this method is suitable for measuring long-lasting cognitive processes such as watching a video (Muthukumaraswamy et al., 2004; Perry and Bentin, 2010; German-Sallo and Ciufudean, 2012; Kwon and Lee, 2020). Through wavelet transformation, the square of the energy value and amplitude corresponding to the alpha rhythm were calculated across the groups and conditions. Then, one-way repeated measure ANOVA using the SPSS (Statistical Package for Social Science) was conducted to examine whether there are statistically significant differences among the conditions (slow-tempo background music, fast-tempo background music, and no background music).

In the ERP analysis, the same time range used in the alpha rhythm analysis was used to measure P300; from the point in which a punch or kick occurs up to 300 ms after that hit or kick. The energy value corresponding to the applicable P300 value, amplitude (μV), was calculated and the calculated value was compared and analyzed for energy values according to each frequency between experimental conditions through one-way repeated ANOVA using SPSS.

## Results

This study examined whether the presence of background music (absence of background music vs. presence of background music) leads to different levels of attentional process among the audience and whether the tempo (slow vs. fast) of background music is associated with different levels of attention. First, a one-way repeated ANOVA was conducted on the degree of alpha-rhythm inhibition associated with the three conditions (no background music (N-M), fast-tempo background music (F-M), slow-tempo background music (S-M)). The statistical analysis suggests that there is a significant difference among the conditions [*F* (2, 40) = 5.745, *p* = 0.006, *η_p_*^2^ = 0.223].

Bonferroni *post-hoc* analysis suggests that there is a significant difference (*p* < 0.05) between no background music (*M* = −0.037 μV^2^, *SD* = 0.022) and fast-tempo background music conditions (*M* = −0.171 μV^2^, *SD* = 0.042). In the fast-tempo background music condition, the alpha rhythm was suppressed to a greater degree compared to the no background music conditions, suggesting that the attentional process of the participants was activated to a greater degree in the fast background music condition. However, no statistically significant difference was found between the no background music and slow-tempo music conditions (*M* = −0.072 μV^2^, *SD* = 0.021; *p* = 0.11). There was also no significant difference between the fast- and slow-tempo background music conditions (*p* = 0.16).

**Figure 2:**
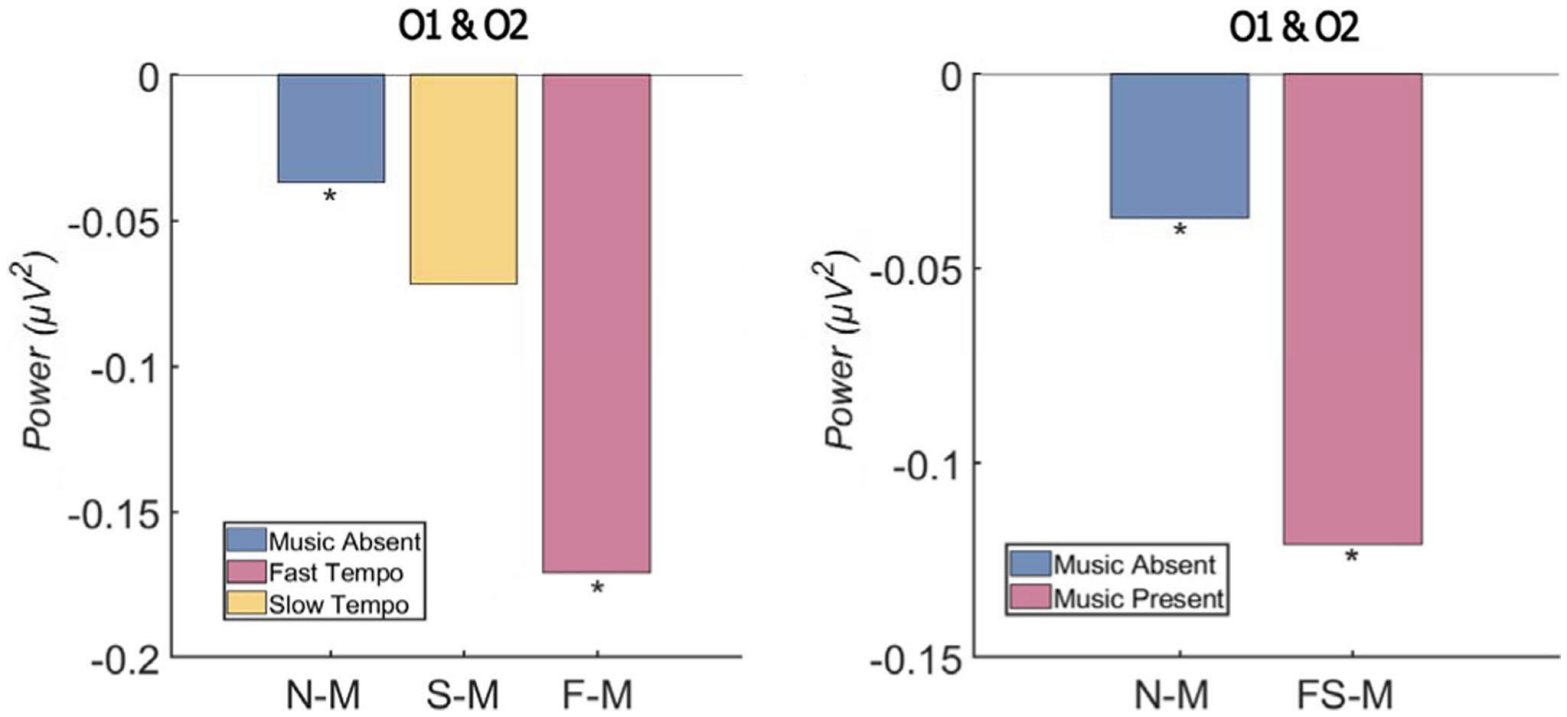
Alpha-rhythm suppression level by condition (^*^denotes a statistically significant difference).

To examine whether the presence of background music leads to different levels of alpha-rhythm suppression, we combined the slow- and fast-tempo conditions (SF-M; *M* = −0.121 μV^2^, *SD* = 0.024) and compared this with the no background music condition. A one-way repeated ANOVA suggests that there is a significant difference between the average value of slow and fast background music conditions and the value of no background music condition [*F* (2, 40) = 5.745, *p* = 0.006, *η_p_*^2^ = 0.223]. This indicates that the conditions with background music altogether were associated with a greater degree of alpha-rhythm suppression thus a greater degree of attentional process, compared to the no background music condition. The alpha-rhythm suppression value for each condition is illustrated in Figure 2.

Second, another one-way repeated ANOVA was performed on P300 to conduct ERP analysis. The ANOVA revealed that there is a statistical difference among the P300 levels of the three conditions [*F* (2,40) = 11.500, *p* = 0.000, *η_p_*^2^ = 0.365]. Bonferroni *post-hoc* analysis indicated that there was a significant difference between the no background music condition (*M* = 0.730 μV^2^, *SD* = 0.044), and fast tempo condition (*M* = 1.015 μV^2^, *SD* = 0.041, *p* < 0.001). Additionally, a difference was observed between the no background music and slow tempo condition (*M* = 0.895 μV^2^, SD = 0.039; *p* < 0.05). However, there was no significant difference between the slow- and the fast-tempo background music conditions (*p* = 0.189).

**Figure 3:**
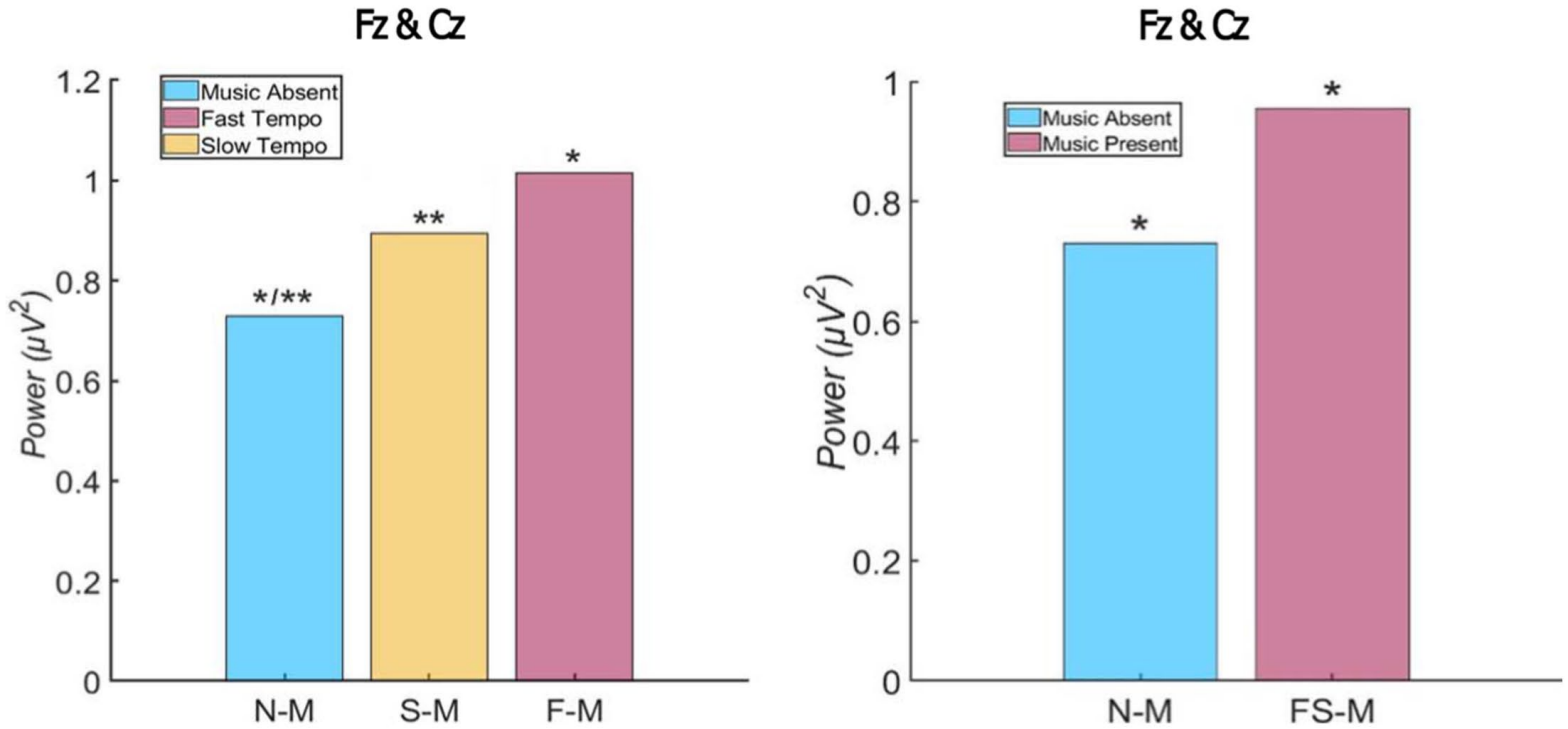
Event-related potential (ERP) inhibition level by condition (^*^, ^**^denote statistically significant differences).

To examine whether P300 levels differ based on the presence of background music, another repeated ANOVA was conducted on the average P300 value between the slow- and fast-background music conditions and the value of no background music condition. The test suggests the P300 level was significantly greater in the background music conditions (slow + fast tempo; *M* = 0.955 μV^2^, *SD* = 0.027) compared to the no background music condition, indicating viewers were more attentive when background music was present than when background music was absent. The P300 value for each condition is illustrated in Figure 3.

## Discussion

The present study examined the impact of background music on the attentional process of a film audience. Particularly, the current study examined whether the presence and tempo of background music can lead to different levels of attention. Two EEG analyses (alpha-rhythm suppression and ERP) that respectively use two different indicators of the attentional process were used.

The analyses suggest that the presence of background music had a positive effect on the audience’s attentional process. The two analyses uniformly showed that greater levels of attentional processes were involved in the conditions with background music (compared to the condition without any background music). This implies that the audience can pay more attention to the visual image of the footage when background music is available. Previous studies examining the relationship between background music and attention have shown inconsistent results. That is, background music enhanced attention in some cases (Allan, 2006; Lavack et al., 2008) but not in others (Wakshlag et al., 1982; Chou, 2010; Shih et al., 2012).

From the perspective of limited capacity theory (Kahneman, 1973), attention is a limited cognitive resource. Therefore, background music during a cognitive activity (e.g., reading) can consume one’s cognitive resources and ultimately reduce one’s attention on the task. Our findings, however, indicate that in a situation in which cognitively demanding tasks are not required, background music can increase attention level, rather than depleting the restricted attentional resources.

We think that this finding may be related to the elevation of the audience’s arousal level. As noted earlier, background music can induce emotional responses from the audience by generating the overall mood of a film (Holman, 2012). A prior study found that the state of being affectively aroused can influence cognitive processes, whereby attention to visual stimuli is enhanced (McConnell and Shore, 2011). From the perspective of affective arousal, the mood changes that resulted from our background music may have heightened the affective arousal levels of the participants and ultimately encouraged their attention on the stimuli scenes.

Our findings also indicate that the tempo did *not* influence the level of attention. There was no statistically significant difference between the slow- and fast-tempo background music conditions either in the degrees of alpha-rhythm inhibition or P300. This null finding somewhat contradicts our prediction and findings from previous studies that different tempos would be associated with engendering different levels of attention. The null findings however should be considered with caution, as our findings are based on a laboratory setting in which the entire viewing environment was artificially manipulated and all musical variables were controlled for. In the real world, the attentional process of an audience is not just influenced by audio-visual stimulations of a few seconds long but by various factors that form the overall viewing experience, such as dramaturgy and storytelling. Therefore, various possibilities should carefully be considered in addressing the effect of tempo.

As suggested by our findings, the tempo of background music may be an insignificant factor in the audience’s attentional process. Musical factors other than the tempo of background music, such as pitch range and tonality, may play a more important role in inducing attention. Alternatively, the tempo may not have a decisive effect by itself but may only work through interaction with other elements. For instance, the tempo may influence the attentional process only in particular conditions in which a fast tempo meets a high range or when the rhythm of the video and the speed of the tempo harmoniously match one another. Future studies should consider these possibilities when further examining the influence of tempo.

Additionally, the tempo of background music used in our study might not have been perceived as fast or slow enough for the participants. The tempo used in the slow and fast background music conditions were *allegro* and *largo*, respectively, which are clearly distinguishable from one another and considered relatively slow and fast according to music theory. However, some participants may not have sufficiently perceived the difference in the tempo, as such judgments can vary across individuals. Future studies therefore may need to consider participants’ individual differences in perceiving different tempos and use a more diverse set of tempos when examining the effect of tempo.

To examine the effect of background music, the present study used two analyses that, respectively, use alpha-rhythm suppression and P300. Most of the findings from the two analyses were identical and offered more robust evidence regarding the impact of background music on the attentional process. One difference however was found among these analyses: while ERP analysis revealed a statistically significant difference in the no-music and slow-tempo music conditions, alpha-rhythm suppression analysis did not. Such a difference in the findings may be due to the different capabilities each analysis is known to offer. Namely, both ERP and alpha-rhythm suppression analyses allow the measuring of a real-time cognitive response to a stimulus, yet ERP is considered a more sensitive tool, as the response is measured particularly at the peak (in 1/1,000 s unit time after the stimulus is presented), as opposed to the alpha-rhythm suppression approach, in which the measurement lasts for 1 s and is then averaged over that whole 1 s. Perhaps the difference in neuro response elicited in the no-background music and slow music conditions may have been extremely small, and the ERP that captured responses at a particular point of time was sensitive enough (more than alpha-rhythm suppression) to detect the difference in these two conditions.

Despite the growing importance of sound in visual media, few neurophysiological studies to date have examined the effect of sound on the audience. This study presents empirical data from an EEG experiment and offers theoretical foundations for the effect of background music on the audience. More research that takes other factors into consideration is needed to provide a more accurate and richer discussion on this topic. However, we hope that the findings of the present study serve as one of the initial data to offer a theoretical base for the effect of background music and further contribute to the production of audience-oriented media content.

## Data availability statement

The data that support the findings of this study are available from the corresponding author, SL, upon reasonable request.

## Ethics statement

The studies involving human participants were reviewed and approved by Young-Min Choi, Dong-A University. The participants provided their written informed consent to participate in this study.

## Author contributions

Y-SK has conceived and designed the analysis, collected the data, performed the analysis, and written the paper. JL has conducted the experiment and written the paper. SL has conceived and designed the analysis, written, reviewed, and edited the paper. All authors contributed to the article and approved the submitted version.

## Funding

This work was supported by the National Research Foundation of Korea (NRF) grant funded by the Korea government (MSIT; No. NRF-2020R1G1A1101384).

## Conflict of interest

The authors declare that the research was conducted in the absence of any commercial or financial relationships that could be construed as a potential conflict of interest.

## Publisher’s note

All claims expressed in this article are solely those of the authors and do not necessarily represent those of their affiliated organizations, or those of the publisher, the editors and the reviewers. Any product that may be evaluated in this article, or claim that may be made by its manufacturer, is not guaranteed or endorsed by the publisher.
